# Behavioral physical activity intervention coupled with standard post-cancer directed treatment care to mitigate chronic pain in childhood cancer survivors: A protocol for a single-center, pilot randomized controlled trial

**DOI:** 10.1016/j.conctc.2023.101216

**Published:** 2023-10-05

**Authors:** Smita Dandekar, Maxime Caru, Kathryn H. Schmitz

**Affiliations:** a Department of Pediatrics, Division of Hematology and Oncology, Pennsylvania State Health Children's Hospital, Hershey, PA, USA; b Department of Public Health Sciences, Pennsylvania State University College of Medicine, Hershey, PA, USA; c Division of Hematology and Oncology, University of Pittsburgh, Pittsburgh, PA, USA

**Keywords:** Exercise, Pediatric cancer, Supportive care, Pain, Integrative oncology

## Abstract

**Background:**

Long term survivors of childhood cancer have a high prevalence of chronic pain. Novel, multidisciplinary approaches to manage pain, are needed to allow for a reduction in the use of opioids for pain management. Physical activity is highly effective in managing chronic pain in children and adolescents, however, evidence about the combination of physical activity intervention and pain medications in chronic pain management in childhood cancer survivors is lacking. The aim of this study is to investigate the feasibility, acceptability and preliminary effects of a behavioral physical activity intervention integrated into standard post cancer directed treatment care to mitigate chronic pain in this unique population.

**Methods:**

This is a single site pilot randomized controlled trial of a 16-week physical activity intervention coupled with standard care. The primary aim is to assess the feasibility and acceptability of the physical activity intervention in childhood cancer survivors with chronic pain. Secondary aims include evaluating the differences in functional and psychosocial outcomes along with self-reported pain scores and cumulative dose of pain medications between the exercise group and standard cancer care group. The physical activity intervention is a home-based program structured to increase patients' physical activity behavior and to favor low intensity bodily movement using aerobic exercise and resistance training.

**Conclusions:**

This study will demonstrate that behavioral supportive measures like physical activity may be a novel means to improve cancer related chronic pain in young survivors of childhood cancer and decrease medication usage for pain along with improvement in functional and psychosocial outcomes.

## Highlights

• Integrating a behavioral PA program into standard post-cancer directed care has a strong potential to mitigate chronic pain in CCS.
• Pilot data from this study will provide essential information to guide PA recommendations for CCS with chronic pain.
• This study will inform the development of clinical care guidelines for multidisciplinary management of chronic pain in CCS.

## 1 Introduction

Chronic pain is defined as persistent or recurrent pain lasting longer than 3 months [1] and affects one in four Americans [2]. Its annual cost ($635 billion) is greater than the annual costs of heart disease ($309 billion) and cancer ($243 billion) combined [3]. In the general pediatric population, chronic pain affects up to 35% of children and adolescents worldwide [4], leading to physical and mental health difficulties [5,6].

In long-term survivors of childhood cancer, the prevalence of chronic pain ranges from 11.0% to 43.9% across studies [7]. Moreover, 1 in 2 children and adolescents diagnosed with cancer who experience chronic pain will progress to adulthood with chronic pain, even 25.5 years after being diagnosed with cancer [8]. Evidence shows that childhood cancer survivors (CCS) are more likely to report pain compared to their healthy peers [9]. Moreover, the frequency of chronic pain increases with age, with a higher prevalence during adolescence as compared to earlier in childhood [10,11] with girls reporting chronic pain more frequently than boys [12,13]. There are several reasons for chronic pain in CCS, such as the long-term adverse effects of cancer and treatments and the invasive procedures they received during their disease trajectory [7,14,15].

Treating chronic pain early on is important because if pain continues into adulthood [8,16], it is often more difficult to treat, as observed in patients without cancer [[17], [18], [19]]. Chronic pain in children and adolescents has been associated with reduced physical function and physical activity (PA) levels, with resultant effects on mental health and psychosocial wellbeing (quality of life, anxiety, depression, fatigue) [5,6,13,20]. While non-opioid medications are used to treat mild to moderate chronic pain, opioid medications are often given to treat moderate to severe chronic pain [21] which can be highly addictive, especially among youth in the United States [22,23]. The proportion of patients diagnosed with cancer who receive opioid pain medication ranges from 6% to 46% across studies.

Multidisciplinary approaches to manage chronic pain are needed to improve the quality of life of the CCS and to reduce the use of opioids which would also have the benefits of improving CCS' physical and psychosocial health [24]. There is growing evidence for the positive effects of non-pharmacological interventions such as PA training on organ function, fatigue and physical well-being in children during and after treatment for cancer [25]. PA has been shown to be a promising intervention to improve fitness, biomarkers of cardiometabolic health, inflammation and adipokines among adult cancer patients and children, adolescent and young adult cancer survivors [26,27]. Changing health behaviors through exercise training could help children and adolescent cancer survivors increase their time out of bed, change their sedentary lifestyles and get them to be more physically active, avoid physical deconditioning from inactivity and lead to improvements in mental and physical health.

We recently hypothesized that integration of PA into the management of patients' chronic pain could be an effective strategy to effectively mitigate pain while simultaneously reducing the use of pain medications [24]. We believe that PA could reframe the pain sensation and improve body sensations along with increasing pain tolerance. A systematic review and meta-analysis reported that conditioned pain modulation is impaired in populations with chronic pain and pain pressure thresholds are reduced [28,29]. It has been postulated that PA can improve pain inhibition through changes in the central nervous system resulting in a decrease in pain sensitivity [30] and reports suggesting the association of exercise training with equal or reduced pain sensitivity have been published [31,32]. Observations have shown that exercise training stimulates regions of the brain to improve the functional capacity of the pain modulatory mechanisms [33].

Given the evidence that exercise training has the potential to mitigate chronic pain through reframing pain sensations and sensitivity, we planned a randomized controlled two arm exercise intervention trial to demonstrate that integration of PA into standard cancer care will improve patient reported chronic pain, decrease the need for medications to manage chronic pain and improve functional and psychosocial outcomes in young survivors of childhood cancer.

## 2 Objectives

The primary objective of this single-center, pilot randomized controlled trial is to determine the acceptability and feasibility of a 16-week behavioral PA intervention coupled with standard post-cancer directed treatment care in children and adolescent survivors of childhood cancer. The secondary objectives are to compare changes in chronic pain levels, cumulative dose of pain medications, physical function, and patient-reported outcomes within-group (CCS receiving behavioral PA intervention coupled with standard post-cancer directed treatment care and CCS receiving standard post-cancer directed treatment care alone) and between the two groups.

## 3 Material and Methods

This manuscript adheres to the Standard Protocol Items: Recommendations for Interventional Trials (SPIRIT) Checklist [34]. The trial was registered on ClinicalTrial.gov (NCT05562193).

### 3.1 Study design

This is a single-site pilot randomized controlled trial where participants will be randomized in a 1:1 ratio to the 16-week behavioral PA intervention coupled with standard post-cancer directed treatment care, or to the standard post-cancer directed treatment care.

### 3.2 Ethical approval

This study will be conducted in accordance with the Declaration of Helsinki. The study protocol was approved by the Penn State University Institutional Review Board (#00020762).

### 3.3 Participants screening

Potential participants will be identified through the Division of Pediatric Hematology and Oncology's Comprehensive Operational Database within the Penn State Health Children's Hospital at the Milton S. Hershey Medical Center, and through Penn State Cancer Institute located in Hershey, Pennsylvania in the United-States. The research coordinator will contact the patient's medical oncologist for medical clearance and approval to approach the patient's family (parents or legal guardians) to present the study. If research staff are not able to contact the patient's medical oncologist to obtain their approval to approach their patients, potential participants will not be approached or enrolled.

### 3.4 Participants eligibility

A total of 20 participants will be enrolled following these inclusion and exclusion criteria.

#### 3.4.1 Inclusion criteria

1) Male and female CCS diagnosed with any type of cancer;
2) Completed all standard/planned cancer treatment and are stable at the time of enrollment;
3) < 1-year post cancer therapy;
4) Have chronic pain (pain will be self-reported by the participant, will have to be ≥ 1 on a scale of 10 and lasting for 3 months or longer);
5) Between 10 and 17 years old at the time of enrollment;
6) Who are and who are not non-ambulatory/wheelchair bound;
7) Parent/legal guardian must be able to speak, read and understand the English language;
8) Parent or legal guardian must be able to provide and understand informed consent;
9) Participants must be able to provide and understand assent;
10) Participants must be able to attend visits at Penn State Health Children's Hospital;
11) Participant and parent/legal guardian must have access to a computer, smartphone or tablet.

#### 3.4.2 Exclusion criteria

1) CCS who have not completed all standard/planned cancer treatment and/or not in complete remission at the time of enrollment;
2) > 1-year post cancer therapy;
3) < 10 years old and >17 years old at the time of enrollment;
4) CCS who have evidence in their medical record of an absolute contraindication to complete any of the physical assessments. This exclusion criterion is at the oncologist's discretion and can be applicable any time during the study. Research staff will be informed of the absolute contraindication when they contact the medical oncologists via secure email or secure messaging through the electronic medical record for approval to approach their patients for the study and for medical clearance.
5) CCS who have history of refractory or recurrent cancer;
6) Participants or their parent/legal guardian who are unable to speak, read, and understand the English language;
7) CCS who are unable to access and complete online questionnaires.

### 3.5 Enrollment and consent/assent procedures

The informed consent document will be reviewed in-person with the potential participants and their parents or legal guardians. All the potential questions related to the study will be answered. All eligible and interested participants will be consented for study participation by an approved study team member. To ensure that children and adolescents (between 10 and 17 years old at the time of enrollment) give assent without parental influence, they will be asked to give a fair explanation of the study without the help of their parents. They will be asked personally if they agree to participate before giving their assent. Written informed consent will be obtained from parents or legal guardians and assent will be obtained from the participants prior to the initiation of any study-related activities.

Participants and their parents or legal guardians will be informed that their participation in the research is voluntary, that their specific information will be de-identified, and that their decision to participate in this research study will not affect their care. Specific language in the consent document will allow to clarify that the relationship with their medical oncologist will not be altered if they choose not to consent to the research or choose to withdraw later. If enrolled participants request withdrawal from the study, we will ask for their withdrawal reasons and no further contact will be made. The participants and their parents or legal guardians will be informed that their data that was already collected can be retained and/or not included in the analysis according to their decision. The request for previously collected data to be removed from the analysis must be provided to the study team in writing.

### 3.6 Randomization process

A computerized random number generator will be performed to randomly assign each participant to the behavioral PA intervention coupled with standard post-cancer directed treatment care group (n = 10) or the standard post-cancer directed treatment care group (n = 10) in the order they are enrolled (randomly, without the ability to predict the next assignment). A staff member not associated with the enrollment of the participants will create an envelope for each of the participants to be enrolled (n = 20). The envelopes will be sealed. Thus, staff members involved in the recruitment will not have any influence on the allocation of the patients.

### 3.7 Behavioral PA intervention

Participants who are randomized to the intervention group will participate in a 16-week behavioral PA intervention coupled with standard post-cancer directed treatment care. The intervention will be based on the Children's Oncology Group Guidelines for Diet and PA recommendations [35], the International Pediatric Oncology Exercise Guidelines [36], and on successful PA interventions ongoing at Penn State Children's Hospital.

The behavioral PA intervention will be structured to increase patients' PA behavior and to favor low intensity bodily movement (e.g., activities expending >1.5 to 3 metabolic equivalent (METS) or intensity <5 on a scale of 0–10), starting with a session duration of <15-min, three days per week. The session duration and frequency will increase over time. Participants will receive a set of resistance bands and a Fitbit (Inspire 3) at the beginning of the intervention, and they will be asked to wear their Fitbit every day (day and night) for the duration of the study. To note, data generated from the Fitbits will not be collected since the purpose of giving a Fitbit to participants is only for them to keep track of their physical activities.

The behavioral PA intervention will also include support calls and text messages from the research staff, as follows: weekly support calls (weeks 1–4), biweekly support calls (weeks 6 and 8) and texts (weeks 5 and 7), monthly support calls (weeks 12 and 16) and texts (weeks 9, 10, 11, 13, 14, 15). The behavioral PA intervention will be individualized according to patients' health status, results from the physical function assessment, and the most recent week's achieved PA. The intervention will be modified and adapted during weekly support calls or text messages, including frequency, intensity, time and type to maximize participants' success. The behavioral PA intervention will be conducted by an exercise physiologist and an exercise expert with specialty training in pediatric oncology.

### 3.8 Standard post-cancer directed treatment care

Participants who are randomized to the intervention or control group will continue to receive standard post-cancer directed treatment care at the time of enrollment and during the study.

Of note, participants who are randomized to the control group will only receive standard post-cancer directed treatment care. At the end of the study, however, these participants will be offered an exercise consult along with physical activity recommendations, a set of resistance bands, and a Fitbit (Inspire 3).

### 3.9 Outcome assessments

All participants (n = 20) enrolled to participate in this study will receive baseline assessments the same day they provided assent, and their parents or legal guardians provided written informed consent. Moreover, they will receive follow-up assessments on weeks 4, 8, 12 and 16, as described in Table 1.

**Table 1:** Outcome assessments over 16 weeks.

	Baseline	Follow-up week 1	Follow-up week 2	Follow-up week 3	Follow-up week 4	Follow-up week 5	Follow-up week 6	Follow-up week 7	Follow-up week 8	Follow-up week 9	Follow-up week 10	Follow-up week 11	Follow-up week 12	Follow-up week 13	Follow-up week 14	Follow-up week 15	Follow-up week 16
**Demographic information**	**I/C**																
**Cancer and treatment information**	**I/C**																
**Chronic pain**	**I/C**								**I/C**								**I/C**
**Pain medications**	**I/C**								**I/C**								**I/C**
**Pain medications (from medical records)**	**I/C**				**I/C**				**I/C**				**I/C**				**I/C**
**Psychosocial outcomes**	**I/C**								**I/C**								**I/C**
**PA behavior**	**I/C**	**I**	**I**	**I**	**I**	**I**	**I**	**I**	**I**	**I**	**I**	**I**	**I**	**I**	**I**	**I**	**I/C**
**Physical function**	**I/C**																**I/C**
Isometric handgrip strength	**I/C**																**I/C**
Aerobic capacity	**I/C**																**I/C**
Physical functioning	**I/C**																**I/C**

### 3.10 Participant-reported demographic information

All participants will self-report their date of birth and gender. They will also describe their racial background, their ethnicity and the highest grade of school they completed at the time of enrollment.

### 3.11 Participant-reported cancer and treatment information

All participants will self-report their age at cancer diagnosis, the duration of their cancer treatment, the time since their treatment ended, their cancer diagnosis, and the treatment regimen they received. If necessary, this information may be extracted from medical records.

### 3.12 Participant-reported chronic pain

All participants will self-report their pain levels using the PedsQL pediatric pain questionnaire which is a symptom-specific instrument for children (8–12 years of age), and adolescents (13–18 years of age) to assess pain in pediatric patients with chronic health conditions [37]. The parent-proxy report format will be used to assess the parent's perceptions of their child's pain.

### 3.13 Patient-reported pain medications

All participants will self-report their pain medication usage using a questionnaire developed by the study team. This questionnaire will record usage of prescribed and over-the-counter medications and will gather information about the use of non-prescription pain medications (e.g., over the counter oral and topical pain medications). Additional questions related to smoking behavior and cannabis/cannabinoids use [[38], [39], [40]] will also be asked and self-reported by all participants. Questions related to therapy to manage their pain (massage therapy, physical therapy, occupational therapy, chiropractic care and behavioral therapy) will also be asked and self -reported by all participants.

### 3.14 Patient-unreported pain medications

For all participants, three other strategies will be used to collect data related to pain medications, as follows.

• Chart review to gather information on prescription pain medication usage through medication reconciliation data. Information will be collected about number of pain medications prescribed, total dosage and length of time prescribed. This review will be conducted by the principal investigator who is a pediatric oncologist.
• The PDMP (Prescription Drug Monitoring Program) will be accessed to gather information on all filled prescriptions for controlled substances within the state of Pennsylvania. This review will be conducted by the principal investigator who is a pediatric oncologist.
• Morphine equivalent dose (MED) will be calculated for each patient after looking at the total daily amount of each opioid the patient is taking. Using the standard conversion factors developed by the CDC, the Morphine milligram equivalent (MME) equates the many different opioids into a standard value that is based on morphine and its potency providing an easily interpretable metric to represent opioid use [41]. This calculation will be conducted by the principal investigator who is a pediatric oncologist.

### 3.15 Participant-reported psychosocial outcomes

All participants will answer psychosocial questions using the PROMIS pediatric questionnaires [42], including self-reported physical function mobility (8-item), anxiety (8-item), depressive symptoms (8-item), fatigue (8-item), peer relationships (8-item), pain interference (8-item), pain intensity (1-item), sleep disturbance (8-items), cognitive function (7-item) and social isolation (8-item). The PROMIS pediatric questionnaire has excellent psychometric properties with a Cronbach's alpha coefficient of α > 0.90 for child self‐report and parent proxy-report [43].

### 3.16 Participant-reported PA behavior

All participants will self-report their PA behavior using the original version of the Godin Leisure Time Exercise Questionnaire (GLTEQ) to assess participants' weekly frequencies of strenuous, moderate and light activities for at least 15 min during a typical 7-day period (a week) [44]. They will also be asked to self-report how often they engage in any regular activities long enough to work up a sweat [44]. The GLTEQ has been widely used in pediatric oncology [45]. All participants will also self-report their weekly PA behavior using a PA questionnaire developed by the study team in order to capture the number of days per week that they are physically active (frequency), the number of minutes they were usually physically active (time), the type of PA they did (type), and the intensity of PA they did (intensity). This questionnaire will also capture their motivation, capability, and opportunities to be more physically active the next week.

### 3.17 Physical function assessments

Maximum voluntary isometric handgrip strength of the forearm muscles will be measured, two times, using a calibrated Jamar hydraulic hand dynamometer. This assessment is a valid and reliable measure that has been reported to be easy for children and adolescents to understand and achieve [46].

Aerobic capacity will be measured using a 6-min walk test (6 MW T) [47]. Participants will be instructed to walk as fast as possible in a hallway for 6 min. Participants' perceived exertion (OMNI scale [48]), heart rate, oxygen saturation, blood pressure, and the covered distance will be measured. An equation to predict cardiorespiratory fitness (_pred_VO2_peak_) will be used [47].

Physical functioning will be measured using two different tests. During the 30-s chair stand test, participants will be instructed to complete as many full sit-to-stands as possible within 30-s. During the “Timed Up and Go” test, participants will be instructed to rise from a chair, walk 3 m (10 feet) around an obstacle, and then walk back to the chair and sit down. These tests will be performed twice. Participants' perceived exertion (OMNI scale [48]) will be measured.

### 3.18 Statistical analysis

Sample size calculations were not required for this single-site, pilot randomized controlled trial. The sample size of 20 is justified due to the limited availability of participants with the specific eligibility criteria being investigated.

Acceptability will be assessed and expressed as a percentage as follows: (number of patients enrolled × 100/number of patients approached).

Feasibility will be assessed by the number of participants randomized to the behavioral PA intervention coupled with standard post-cancer directed treatment care who complete more than 50% of the intervention calculated and expressed as a percentage, as follows: (number of interventions completed × 100/number of interventions planned). To note, the planned interventions are the support calls and text messages, as follows: weekly support calls (weeks 1–4), biweekly support calls (weeks 6 and 8) and texts (weeks 5 and 7), monthly support calls (weeks 12 and 16) and texts (weeks 9, 10, 11, 13, 14, 15).

The effects of the behavioral PA activity intervention coupled with standard post-cancer directed treatment care on secondary outcomes (total self-reported pain score, cumulative dose of pain medications, functional and psychosocial outcomes) will be explored using Repeated Measures Analysis of Variance (RM-ANOVA). RM-ANOVA [49] will be used to analyze both within-group and between-group differences in intervention effects. Cohen's *d* effect sizes [50] will be reported to quantify the magnitude of differences over time and to interpret the practical significance of the findings.

Drop-out and missing data will be accounted for and reported. If the reason is an adverse event during the intervention, it will be reported as well. In case of dropouts or missing data, an intention-to-treat analysis will be performed.

### 3.19 Rigor, Reproducibility and data management

To ensure high quality data collection, written standard operating procedures will be created by the principal investigator (SD) and co-investigators (MC and KS) and disseminated to the study team members. When possible, validated questionnaires and measures were chosen. All data will be managed and stored in Research Electronic Data Capture (REDCap).

### 3.20 Data and safety Monitoring

Safety data including adverse events or serious adverse events will be collected through the Pediatric Module of the Patient-Reported Outcomes version of the Common Terminology Criteria for Adverse Events (Ped-PRO-CTCAE). The Ped-PRO-CTCAE will be used to monitor adverse events in both groups at baseline, 4, 8, 12 and 16-week follow-ups. The NIH NCI Ped-PRO-CTCAE form Builder to build a study-specific custom form related to the behavioral PA intervention to provide a more specific scope of the safety of the intervention. The form has been developed in collaboration with and reviewed by pediatric oncologists. The recall period for the Ped-PRO-CTCAE is the past 7 days. The Ped-PRO-CTCAE responses are scored from 0 to 4. Adverse events reported during the study procedure that are different from those assessed at baseline will be reported to the responsible IRB.

The study will be monitored by the Data and Safety Monitoring Committee (DSMC) and will require a review every 6 months and an audit yearly.

### 3.21 Dissemination

The results generated from this study will be analyzed, reported and disseminated through peer-reviewed publications, presentations at conferences focused on the supportive care of patients in oncology, exercise-related meetings and academic conferences.

### 3.22 STRENGHTS and Limitations

Findings from this study will document the acceptability and feasibility of a 16-week PA intervention coupled with standard post-cancer directed treatment care in children and adolescent survivors of childhood cancer, with chronic pain. Although our target sample size is small, pilot data from this study has the strong opportunity to demonstrate that the integrating a behavioral PA intervention into standard post-cancer directed treatment care will contribute to decreasing patient-reported chronic pain, decrease the need for pain medications, and improve functional and psychosocial outcomes in CCS.

Assuming this study is successful, our results will provide a compelling rationale to further examine the integration of behavioral PA interventions to mitigate chronic pain level from both a functional and quality of life perspective. Moreover, exploratory mechanism outcomes will have the potential to be explored, such as pain sensation, body sensation, and pain tolerance in CCS with chronic pain. The central hypothesis would be that exercise training may have the effect of decreasing pain by reframing the pain sensation and improving body sensations, along with increasing pain tolerance.

The literature is scarce regarding the potential of integrating PA into standard post-cancer directed care in CCS. It is possible that barriers such as fear of movement, which would limit the potential of promoting PA behavior changes. Future research in this area is greatly needed.

## 4 Conclusion

There is limited evidence on how PA can impact patient reported chronic pain, need for pain medications, physical function and psychosocial outcomes in young survivors of childhood cancer. Given the great morbidity associated with the late effects of cancer therapy, the proposed study will provide essential information to guide PA recommendations for CCS with chronic pain and could inform the development of clinical care guidelines and policy making decisions for the multidisciplinary management of chronic pain in CCS.

Children and adolescents diagnosed with cancer are rare overall and the design of exercise oncology clinical trials in this population is practically challenging. A strength of our study is the inclusion of CCS diagnosed with any cancer type, though we won't have enough statistical power to perform subgroup analysis, as its a pilot study. Our pilot randomized controlled trial will help us gather data to progress toward a fully powered efficacy trial where specific cancer subtypes could be studied, assuming that the results from this current study justify doing so. Our long-term goal is to identify CCS with chronic pain that could benefit the most from our behavioral PA intervention. At this stage, we can only hypothesize that integration of a behavioral PA intervention will be an effective strategy to mitigate pain while simultaneously reducing the use of pain medications.

In recognition of the urgent need to improve the management of chronic pain, the NIH recently issued a call for projects and announced increased funding for pain research and its “Helping to End Addiction Long-term (HEAL)" initiative to speed scientific solutions to the national opioid public health crisis. Our study is in line with the research funding plan from the NIH and places us at a unique vantage point to build an outstanding and successful research project that answers the NIH call and improves supportive care for CCS.

## Authors' contributions

SD wrote and obtained the grant funding. MC, SD and KS conceptualized and designed the protocol. KS provided input into the development of the protocol. SD and MC drafted the manuscript and KS reviewed the manuscript. All authors have reviewed and approved the final version of the manuscript.

## Declaration of competing interest

The authors declare that they have no known competing financial interests or personal relationships that could have appeared to influence the work reported in this paper.
